# Supplementary material for: Fluctuations in quality of life and immune responses during intravenous immunoglobulin infusion cycles
Source: PLoS One. 2022 Mar 22;17(3):e0265852. doi: 10.1371/journal.pone.0265852 (PMC8939786; doi:10.1371/journal.pone.0265852)

Fig S1. Modified SF-12 and Visual Analog Scale

Subject ID: ______________________________

Visual Date: ______________________________

Instructions for Completing the Questionnaire

Please answer every question. Some questions may look like others, but each one is different. Please take the time to read and answer each question carefully by filling in the bubble that best represents your response.

Your Health in General

In the past week, did you have any infection? Yes No

If yes, what kind? ______________________________

Did you see a doctor/nurse? Yes No

Did you receive treatment? Yes No

Did you have flu-like symptoms: Yes No

Did you have loose stools? Yes No

1. In general, would you say your health is:

Excellent	Very good	Good	Fair	Poor
○ 1	○ 2	○ 3	○ 4	○ 5

2. Compared to the week before, how would you rate your health in general now?

Much better now than one week ago	Somewhat better now than one week ago	About the same as one week ago	Somewhat worse now than one week ago	Much worse now than one week ago
○ 1	○ 2	○ 3	○ 4	○ 5

3. During the past 1 week, how much of the time have you had any of the following problems with your regular daily activities as a result of your physical health?

	All of the time	Most of the time	Some of the time	A little of the time	None of the time
a) Cut down on the **amount of time** you spent on work or other activities	○ 1	○ 2	○ 3	○ 4	○ 5
b) **Accomplished less** than you would like	○ 1	○ 2	○ 3	○ 4	○ 5
c) Were limited in the **kind** of work or other activities	○ 1	○ 2	○ 3	○ 4	○ 5
d) Had **difficulty** performing the work or other activities (for example, it took extra effort)	○ 1	○ 2	○ 3	○ 4	○ 5

4. During the past 1 week, to what extent has your physical health interfered with your normal social activities with family, friends, neighbors, or group?

Not at all	Slightly	Moderately	Quite a bit	Extremely
○ 1	○ 2	○ 3	○ 4	○ 5

5. During the past week, did you suffer from fatigue?

Not at all	Slightly	Moderately	Quite a bit	Extremely
○ 1	○ 2	○ 3	○ 4	○ 5

6. How TRUE or FALSE is each of the following statements for you for the past month?

	Definitely true	Mostly true	Don't know	Mostly false	Definitely false
a) I seem to get sick a little easier than other people	○ 1	○ 2	○ 3	○ 4	○ 5
b) I am as healthy as anybody I know	○ 1	○ 2	○ 3	○ 4	○ 5
c) I expect my health to get worse	○ 1	○ 2	○ 3	○ 4	○ 5
d) My health is excellent	○ 1	○ 2	○ 3	○ 4	○ 5

To help people say how good or bad a health state is, we have drawn a scale (rather like a thermometer) on which the best state you can imagine is marked 100 and the worst state you can imagine is marked 0.

We would like you to indicate on this scale how good or bad your own health is today, in your opinion. Please do this by drawing a line from the box below to whichever point on the scale indicates how good or bad your health state is.

Your own health state today

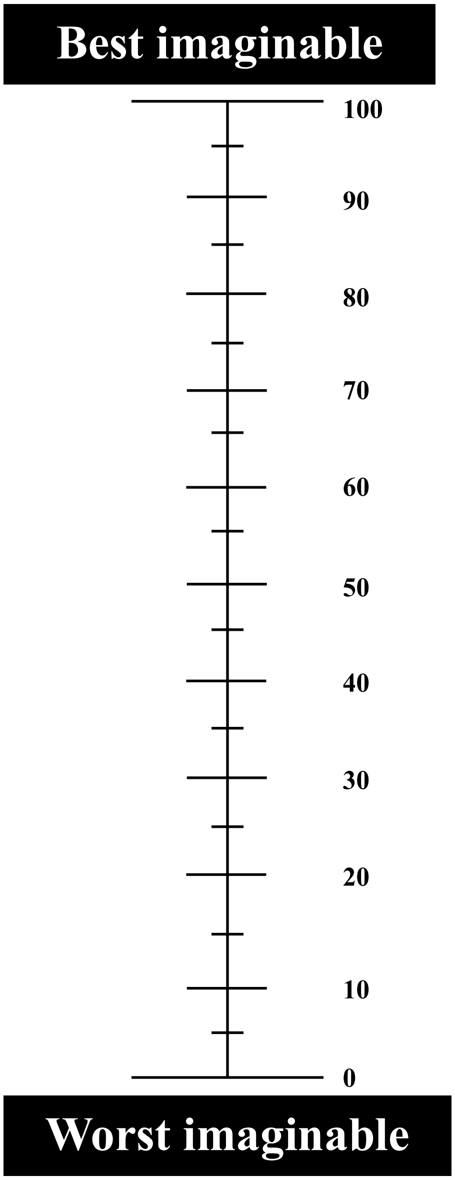

Supplement: S1 Fig — (PDF) [file pone.0265852.s001.pdf]
