# Supplementary figures and images for: Fluctuations in quality of life and immune responses during intravenous immunoglobulin infusion cycles
Source: PLoS One. 2022 Mar 22;17(3):e0265852. doi: 10.1371/journal.pone.0265852 (PMC8939786; doi:10.1371/journal.pone.0265852)

## Slide 1
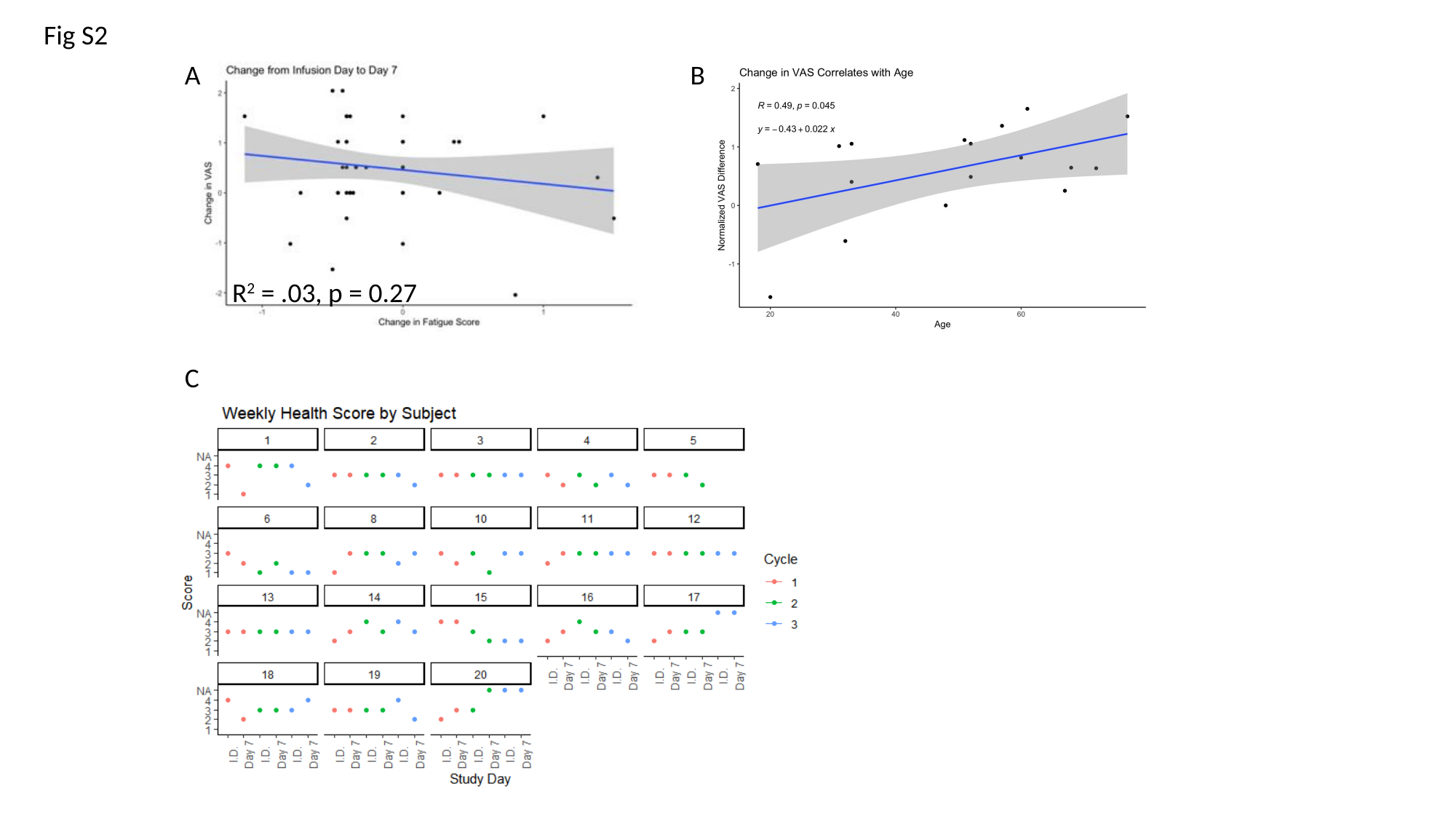

Fig S2
A
B
R2 = .03, p = 0.27
C

Supplement: S2 Fig — (PPTX) [file pone.0265852.s002.pptx]

## Slide 1
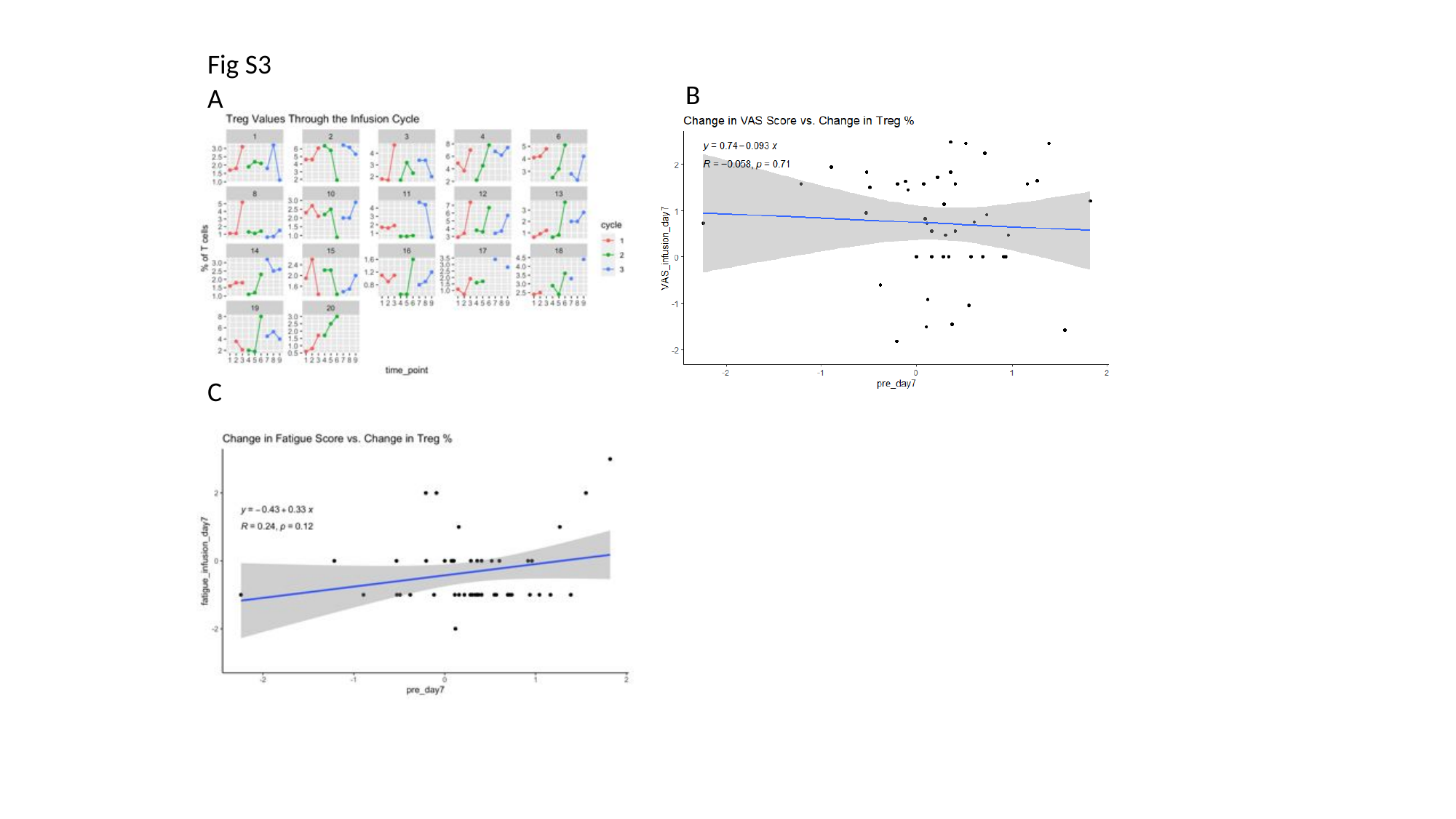

Fig S3
B
A
C

Supplement: S3 Fig — (PPTX) [file pone.0265852.s003.pptx]

## Slide 1
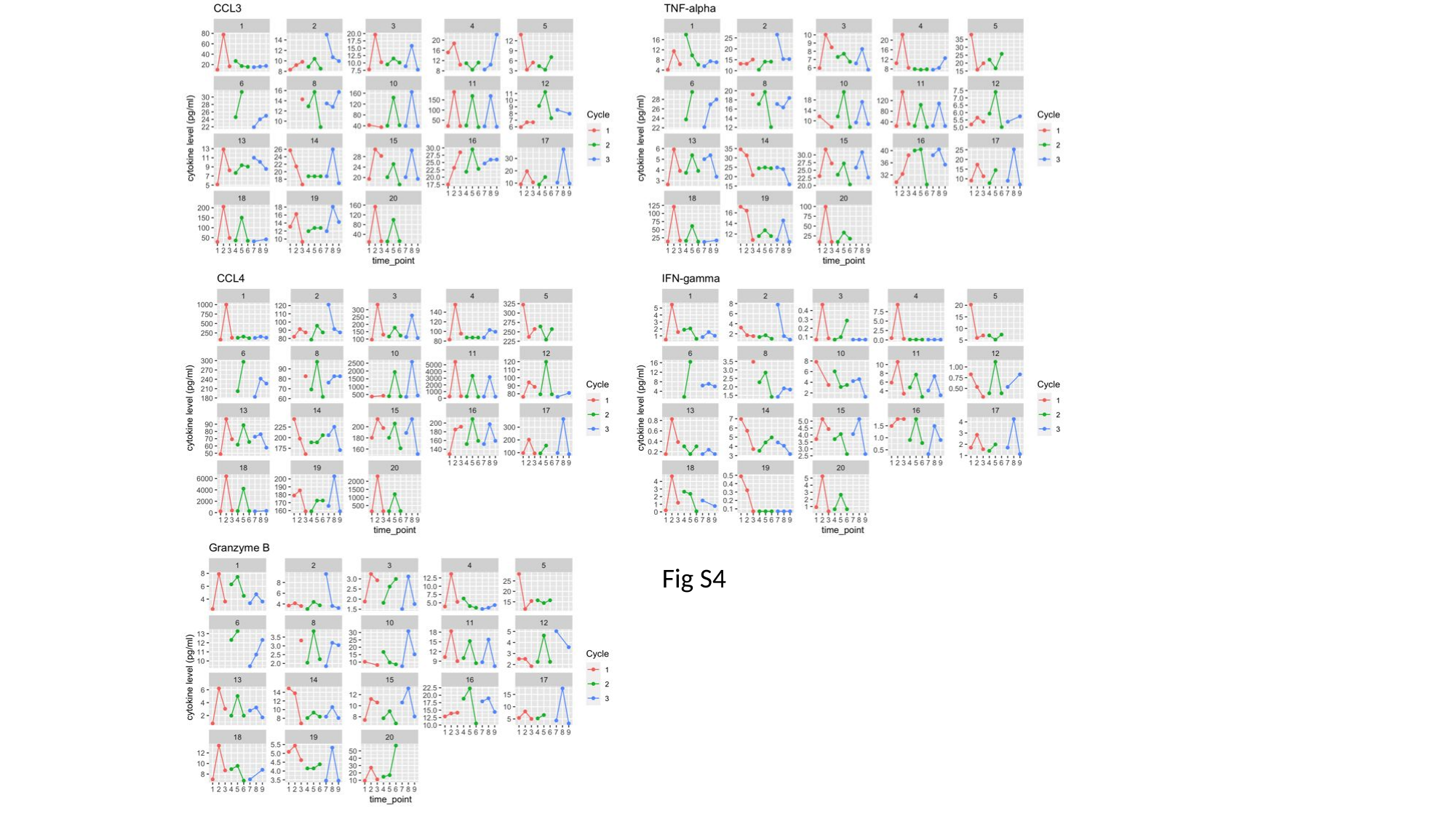

Fig S4

Supplement: S4 Fig — (PPTX) [file pone.0265852.s004.pptx]

## Slide 1
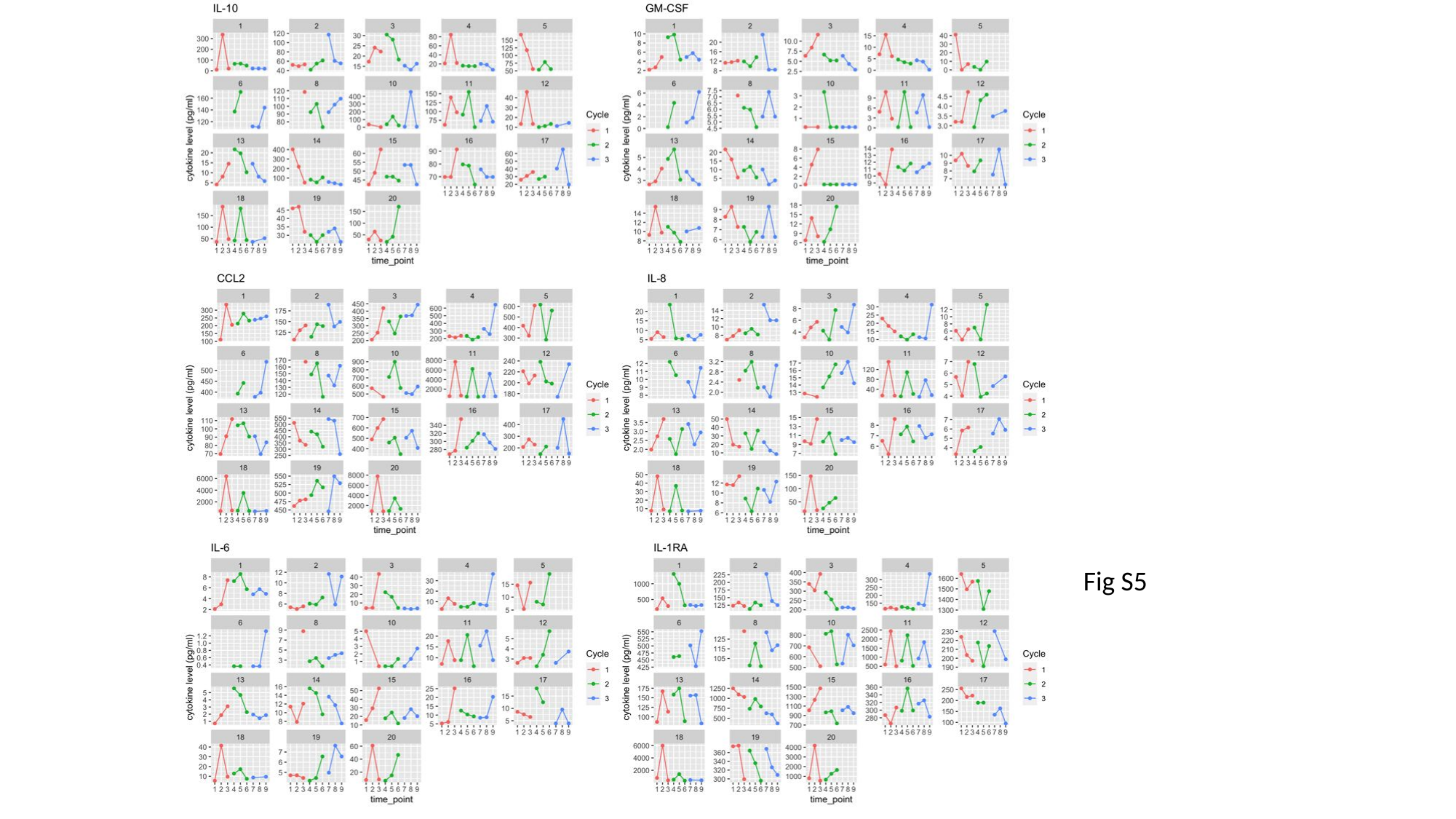

Fig S5

Supplement: S5 Fig — (PPTX) [file pone.0265852.s005.pptx]

## Slide 1
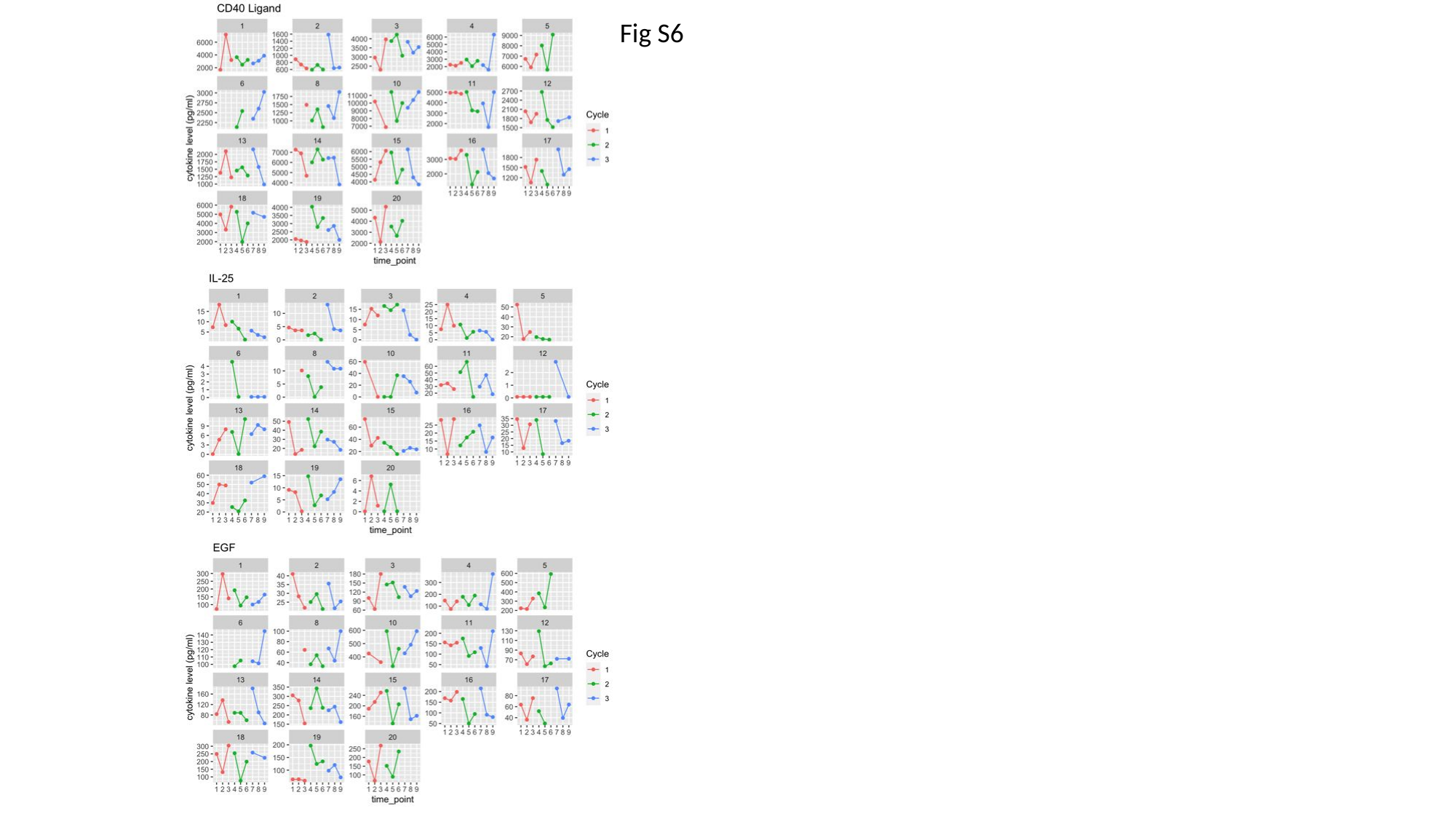

Fig S6

Supplement: S6 Fig — (PPTX) [file pone.0265852.s006.pptx]

## Slide 1
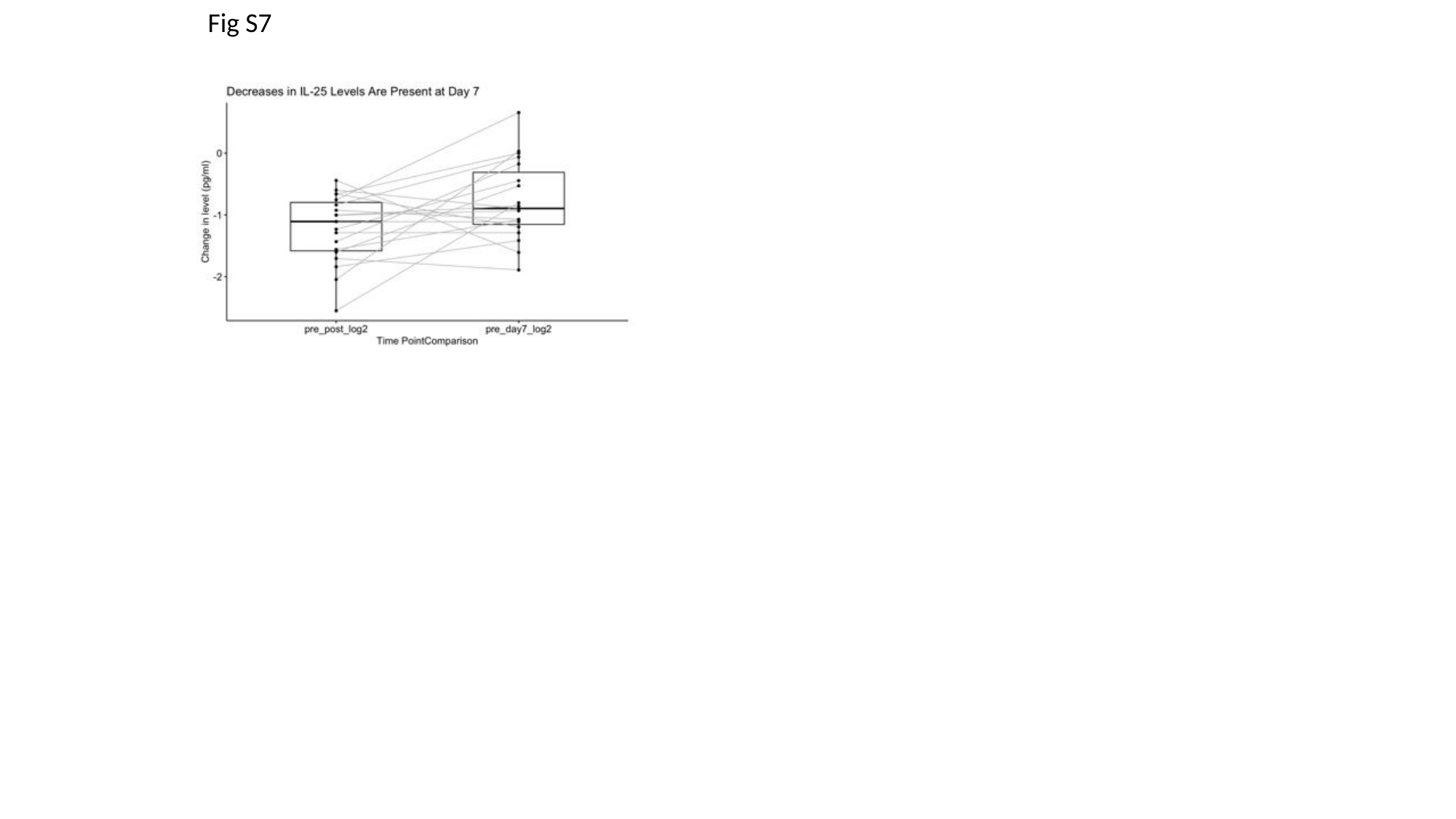

Fig S7

Supplement: S7 Fig — (PPTX) [file pone.0265852.s007.pptx]

## Slide 1
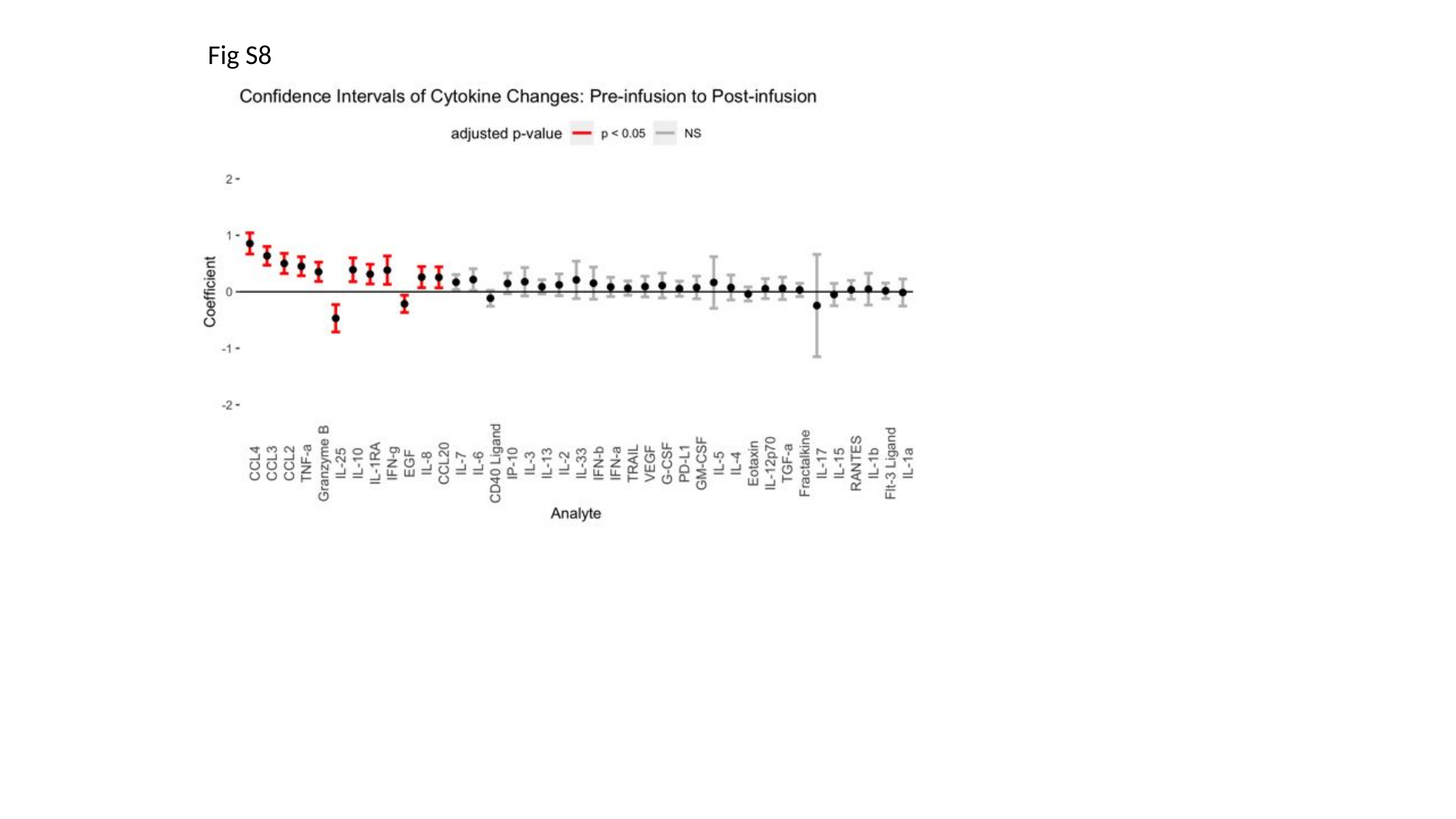

Fig S8

Supplement: S8 Fig — (PPTX) [file pone.0265852.s008.pptx]

## Slide 1
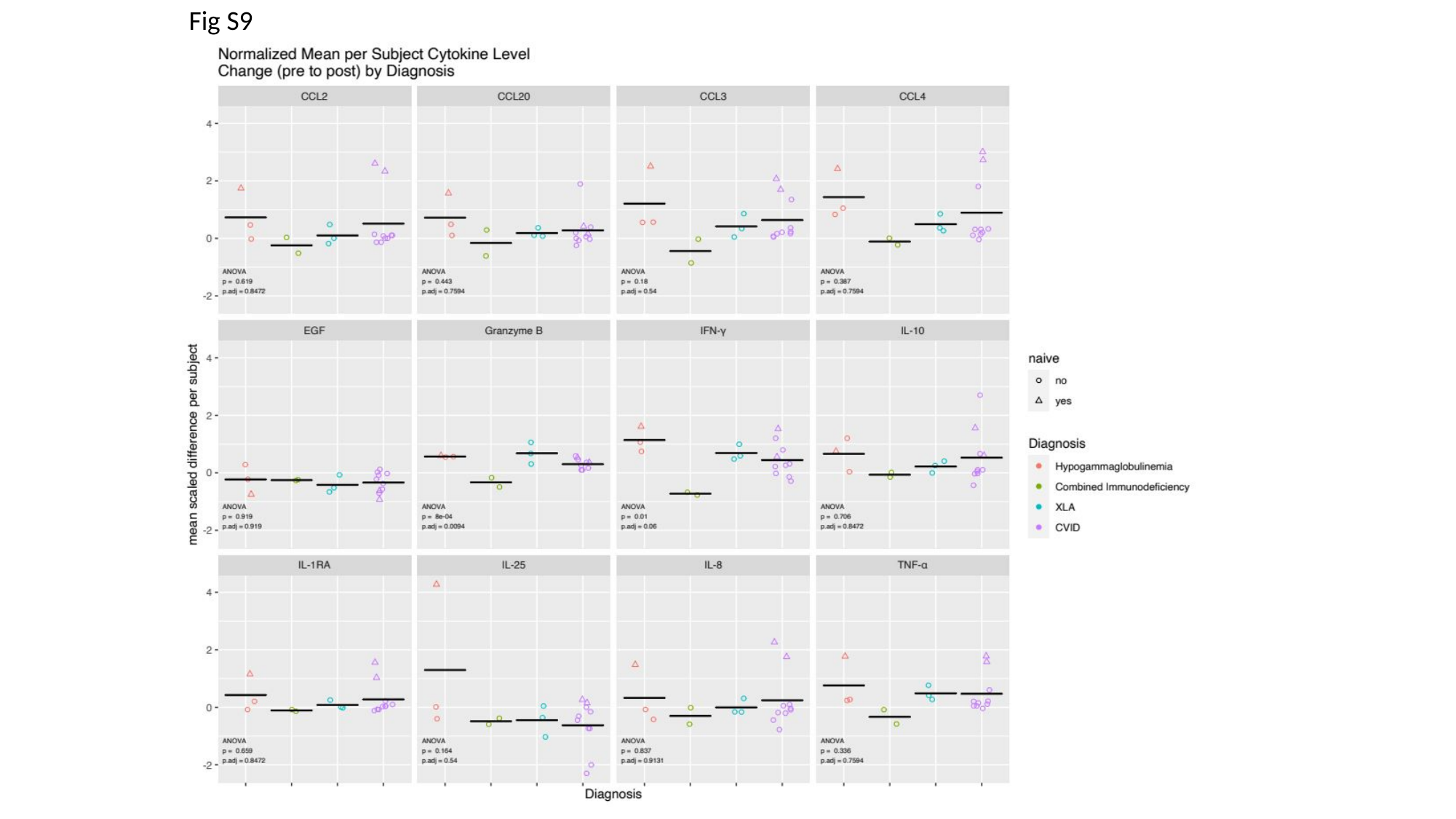

Fig S9

Supplement: S9 Fig — (PPTX) [file pone.0265852.s009.pptx]

## Slide 1
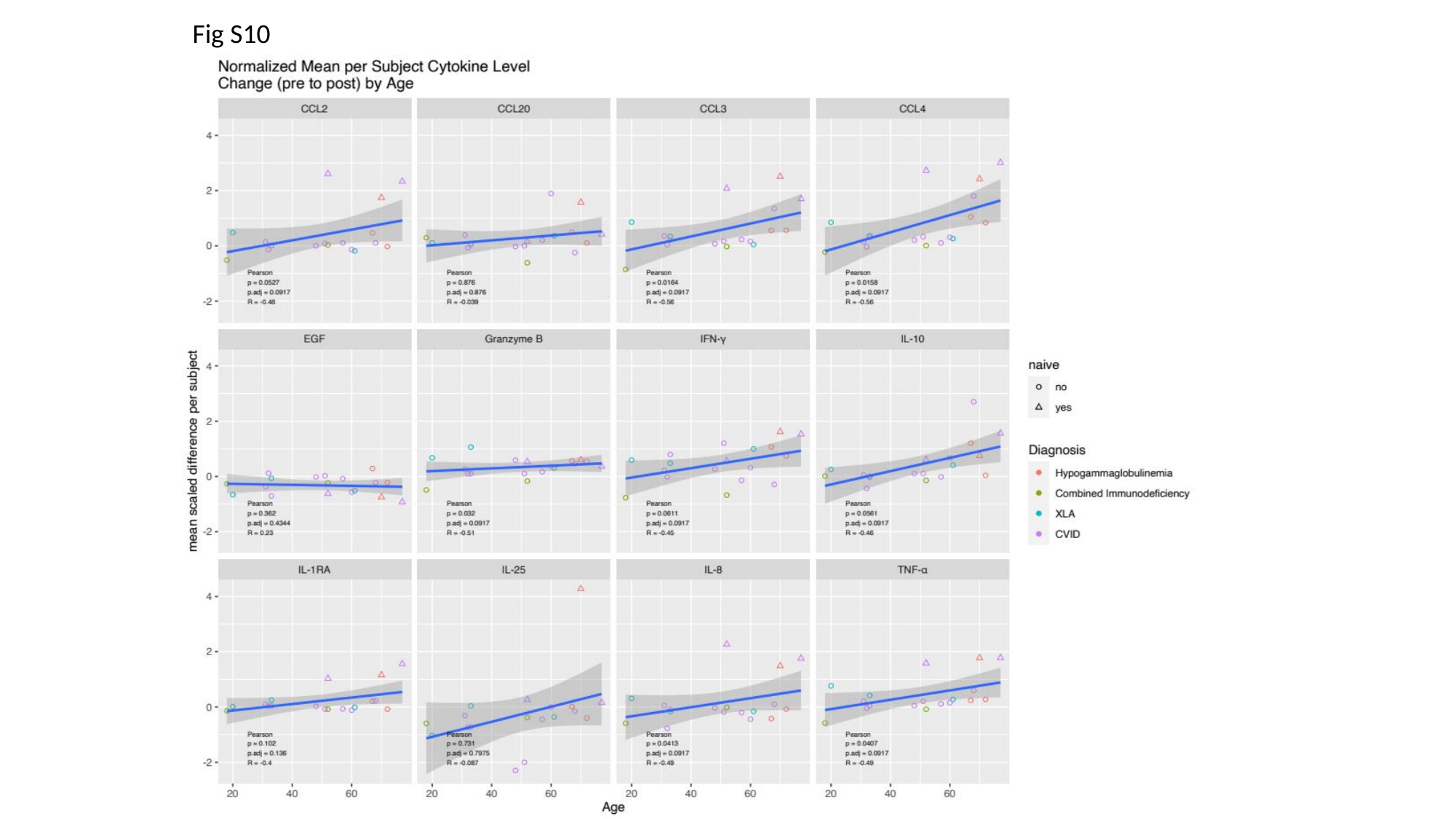

Fig S10

Supplement: S10 Fig — (PPTX) [file pone.0265852.s010.pptx]

## Slide 1
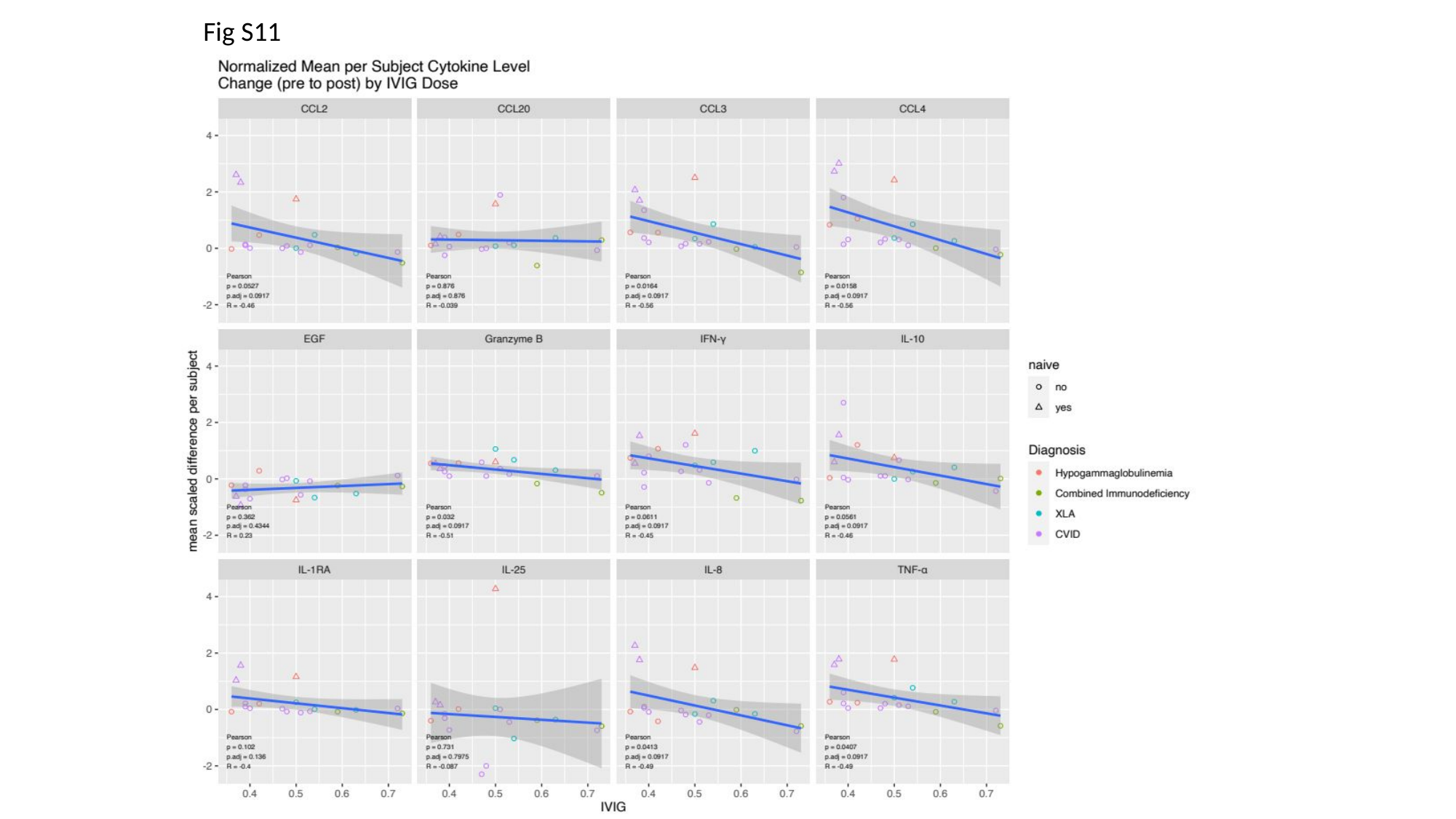

Fig S11

Supplement: S11 Fig — (PPTX) [file pone.0265852.s011.pptx]

## Slide 1
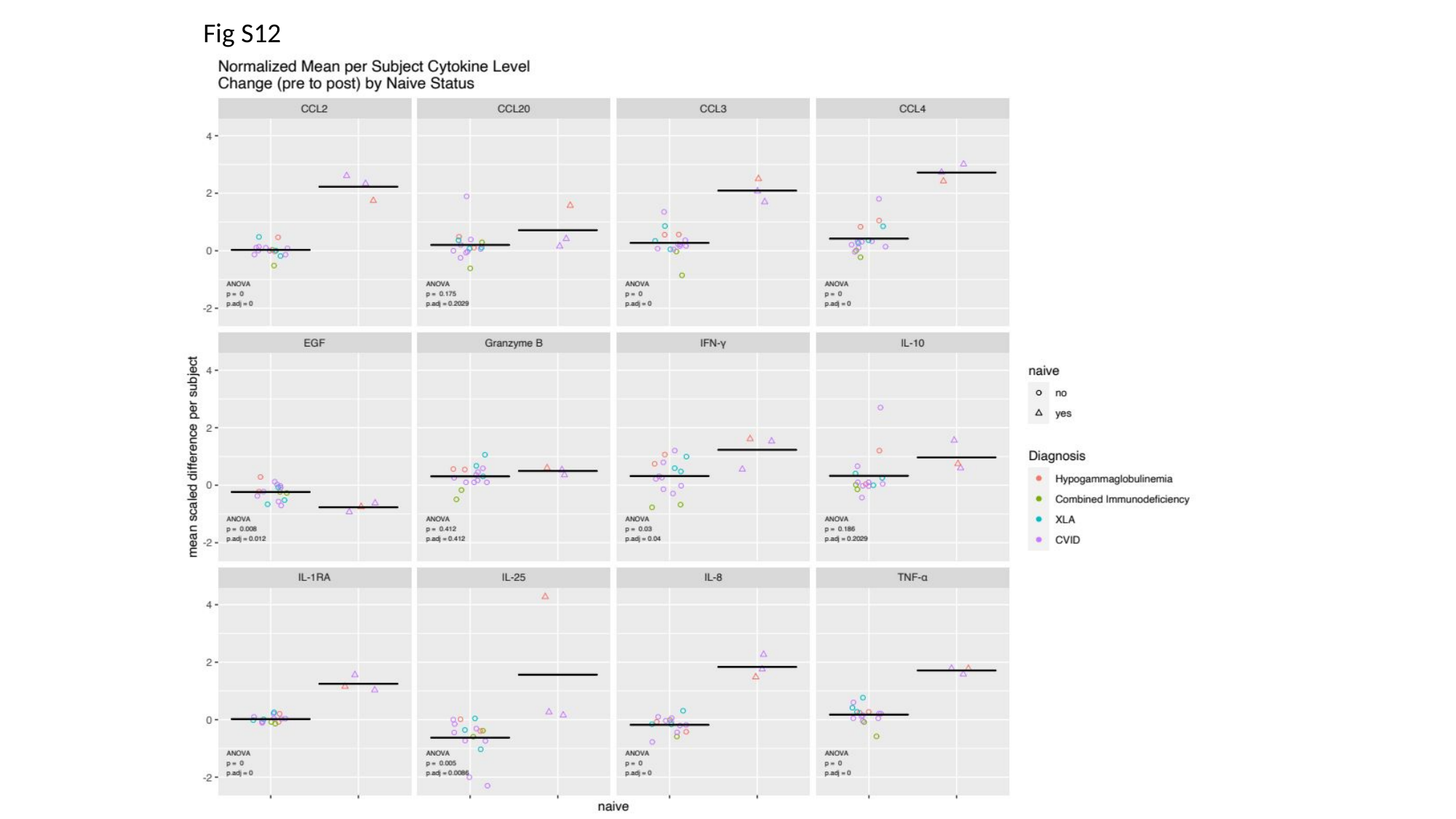

Fig S12

Supplement: S12 Fig — (PPTX) [file pone.0265852.s012.pptx]

## Slide 1
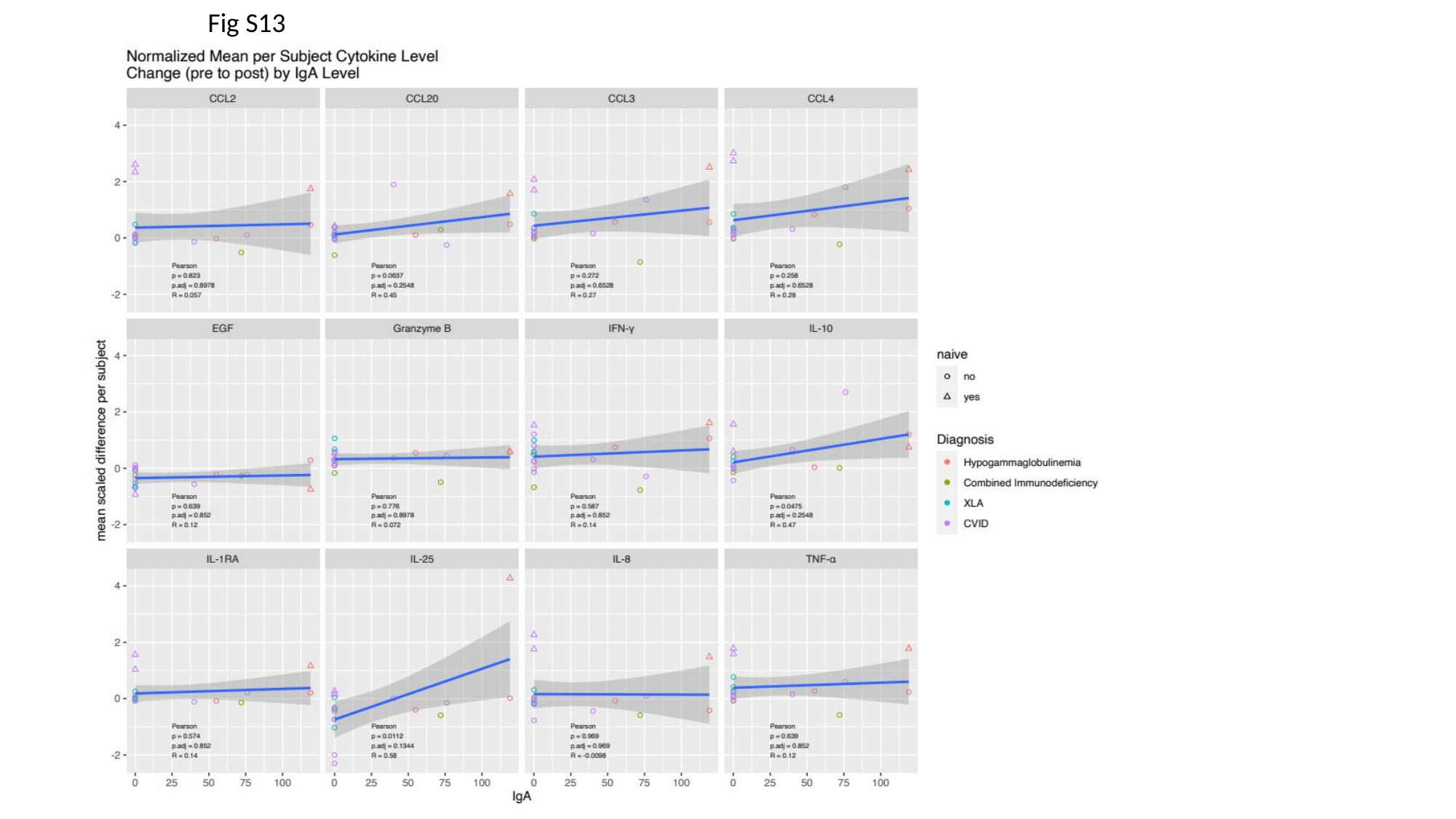

Fig S13

Supplement: S13 Fig — (PPTX) [file pone.0265852.s013.pptx]

## Slide 1
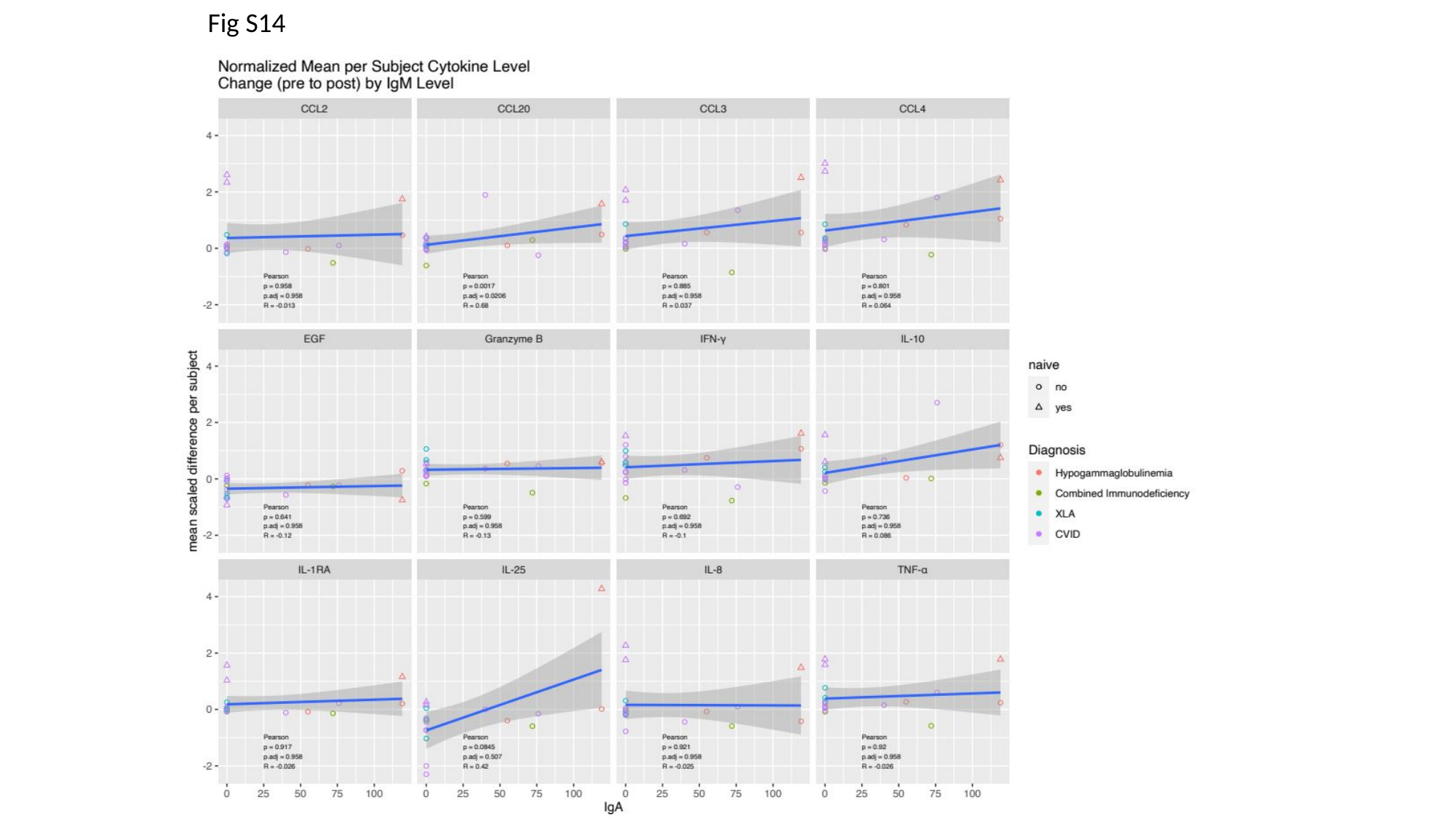

Fig S14

Supplement: S14 Fig — (PPTX) [file pone.0265852.s014.pptx]

## Slide 1
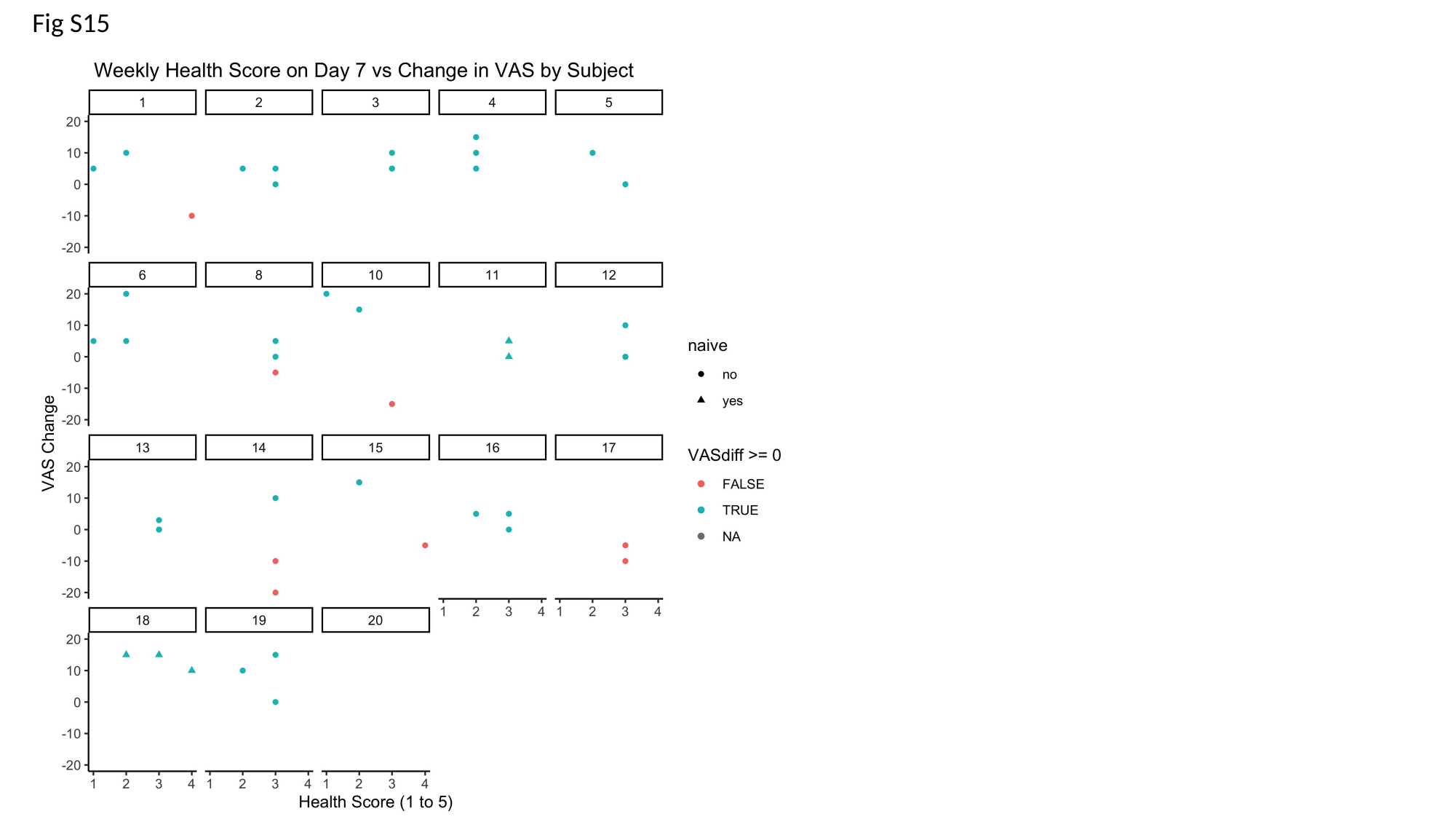

Fig S15

Supplement: S15 Fig — (PPTX) [file pone.0265852.s015.pptx]

## Slide 1
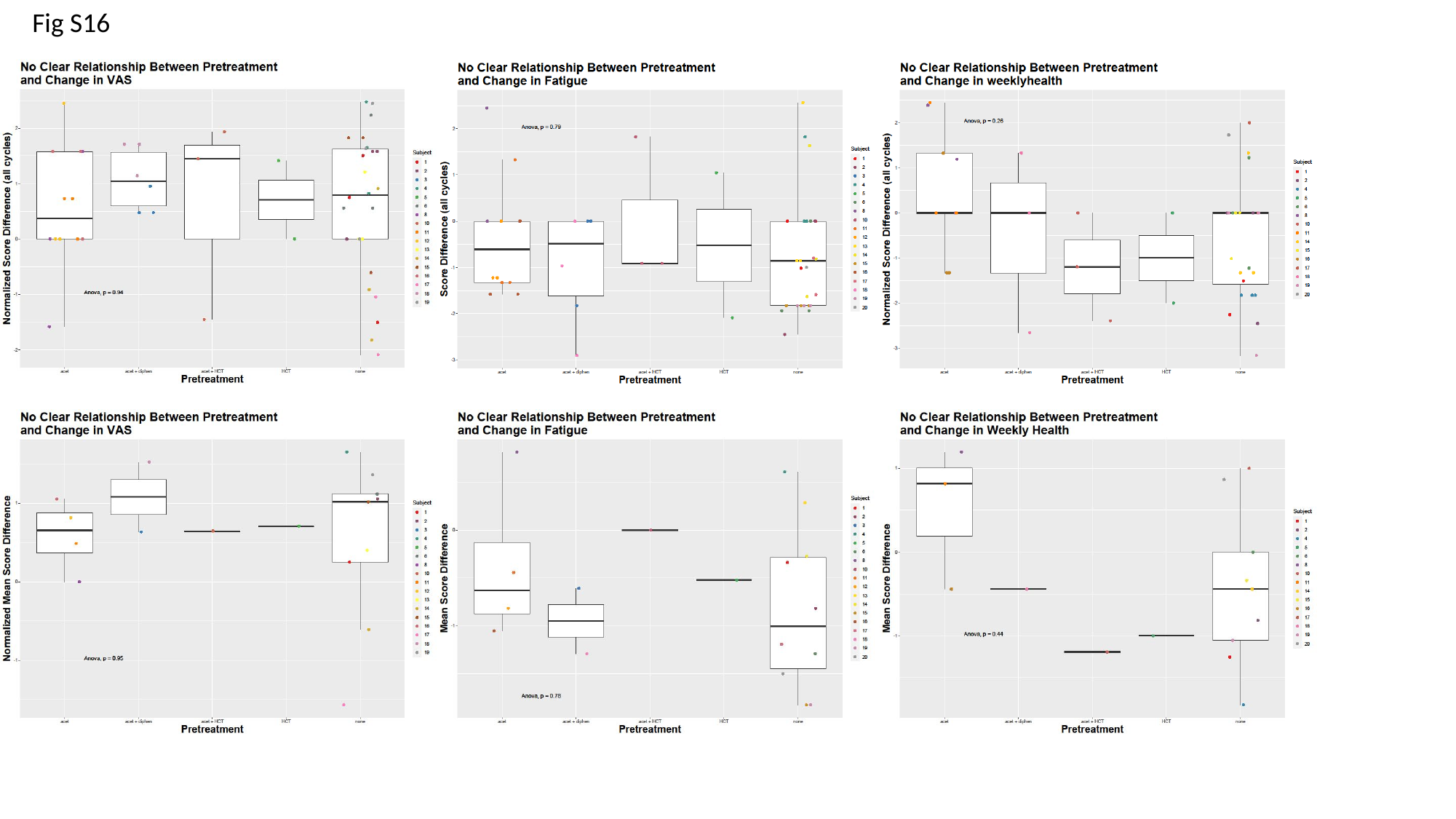

Fig S16

Supplement: S16 Fig — (PPTX) [file pone.0265852.s016.pptx]

## Slide 1
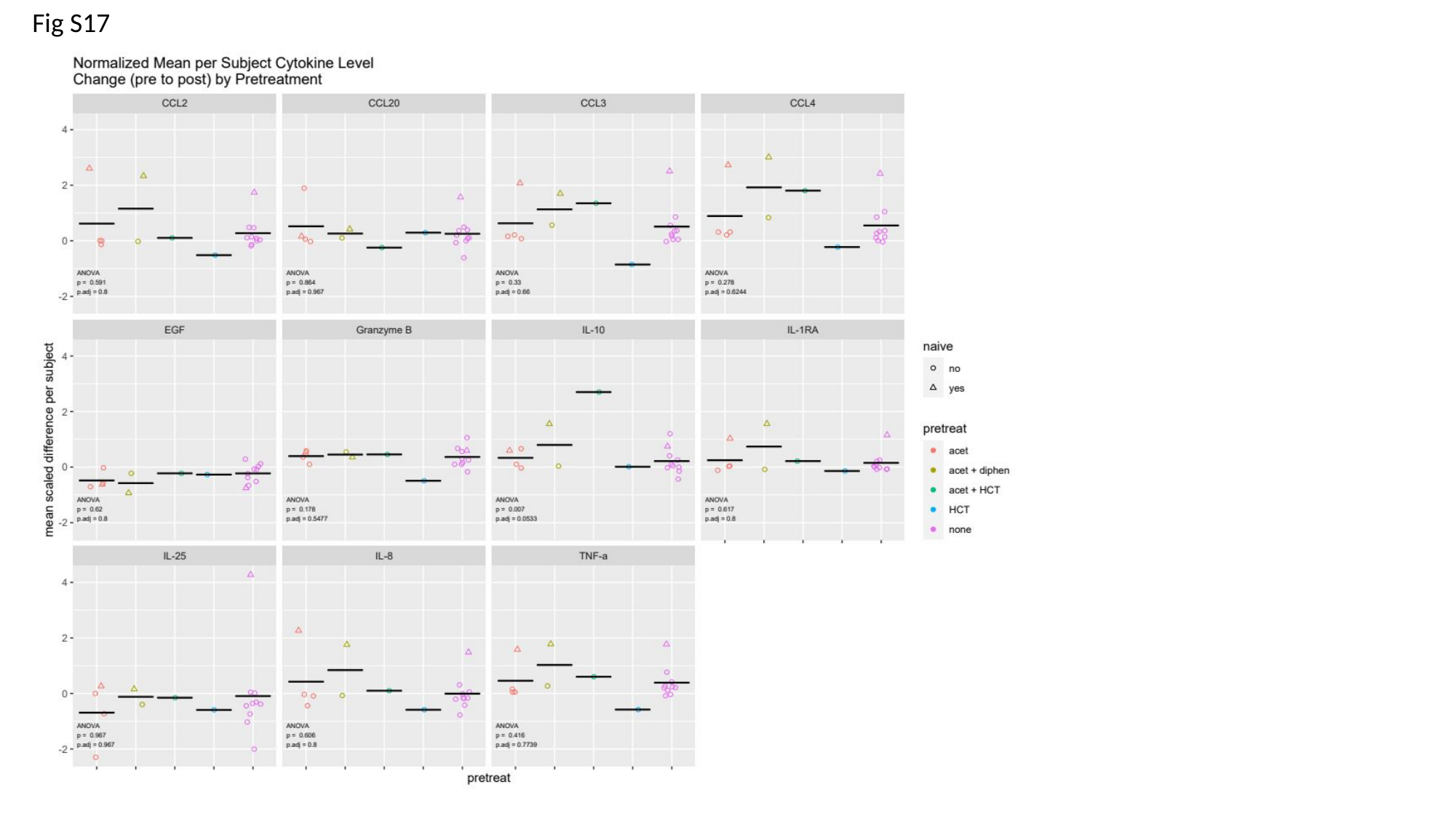

Fig S17

Supplement: S17 Fig — (PPTX) [file pone.0265852.s017.pptx]

## Slide 1
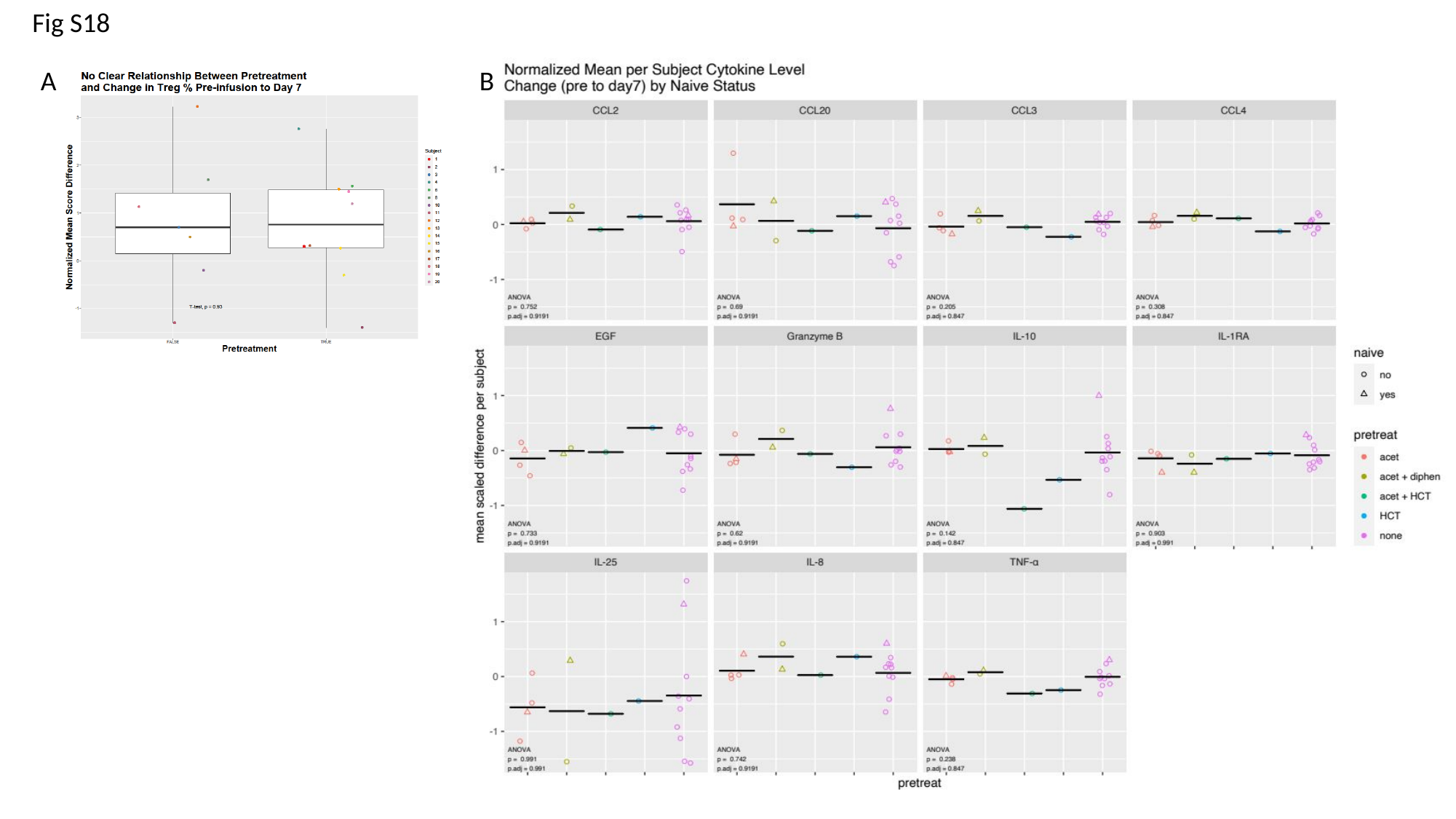

Fig S18
A
B

Supplement: S18 Fig — (PPTX) [file pone.0265852.s018.pptx]
